# Points to consider for sharing variant-level information from clinical genetic testing with ClinVar

**DOI:** 10.1101/mcs.a002345

**Published:** 2018-02

**Authors:** Danielle R. Azzariti, Erin Rooney Riggs, Annie Niehaus, Laura Lyman Rodriguez, Erin M. Ramos, Brandi Kattman, Melissa J. Landrum, Christa L. Martin, Heidi L. Rehm

**Affiliations:** 1 Laboratory for Molecular Medicine, Partners HealthCare Personalized Medicine, Cambridge, Massachusetts 02139, USA;; 2 Autism & Developmental Medicine Institute, Geisinger, Danville, Pennsylvania 17837, USA;; 3 National Human Genome Research Institute, National Institutes of Health, Bethesda, Maryland 20894, USA;; 4 National Center for Biotechnology Information, National Library of Medicine, National Institutes of Health, Rockville, Maryland 20894, USA;; 5 Brigham & Women's Hospital and Harvard Medical School, Boston, Massachusetts 02115, USA

## Abstract

Data sharing between laboratories, clinicians, researchers, and patients is essential for improvements and standardization in genomic medicine; encouraging genomic data sharing (GDS) is a key activity of the National Institutes of Health (NIH)-funded Clinical Genome Resource (ClinGen). The ClinGen initiative is dedicated to evaluating the clinical relevance of genes and variants for use in precision medicine and research. Currently, data originating from each of the aforementioned stakeholder groups is represented in ClinVar, a publicly available repository of genomic variation, and its relationship to human health hosted by the National Center for Biotechnology Information at the NIH. Although policies such as the 2014 NIH GDS policy are clear regarding the mandate for informed consent for broad data sharing from research participants, no clear guidance exists on the level of consent appropriate for the sharing of information obtained through clinical testing to advance knowledge. ClinGen has collaborated with ClinVar and the National Human Genome Research Institute to develop points to consider for clinical laboratories on sharing de-identified variant-level data in light of both the NIH GDS policy and the recent updates to the Common Rule. We propose specific data elements from interpreted genomic variants that are appropriate for submission to ClinVar when direct patient consent was not sought and describe situations in which obtaining informed consent is recommended.

## INTRODUCTION

The benefits of genomic data sharing (GDS), including the potential to improve clinical interpretation of genomic variants and advance genomic medicine, are widely recognized, and the practice has been endorsed by both professional societies and funding agencies (American Medical Association 2013; National Institutes of Health 2014; National Society of Genetic Counselors 2015; ACMG Board of Directors 2017). One of the aims of the National Institutes of Health (NIH)-funded Clinical Genome Resource (ClinGen) (Rehm et al. 2015) is to create a publicly available knowledge base of clinically relevant genes and variants for use in precision medicine and research. As shared genomic data help facilitate this effort, ClinGen is actively involved in assisting laboratories, clinicians, researchers, and patients (Kirkpatrick et al. 2015; Rehm et al. 2015) to submit interpreted variant data to the publicly available ClinVar database of the National Center for Biotechnology Information (NCBI), a repository of human genomic variation and its relationship to human health (Landrum et al. 2014, 2016).

In the ClinVar submission documentation (https://www.ncbi.nlm.nih.gov/clinvar/docs/submit/), the onus is put on submitters to ensure that they have obtained the proper consents and permissions to publicly share data. For research submitters, particularly those supported by NIH funding, this process is relatively straightforward and often required. In 2014, NIH issued its Genomic Data Sharing (GDS) Policy (National Institutes of Health 2014), which expects explicit informed consent be obtained from research study participants for future use of their genomic data for broad data sharing. This policy focuses on those research studies generating large-scale genomic data, such as those with sequence data on more than one gene from more than 1000 participants.

For clinical laboratories, who are often submitting variants identified as the result of clinical genetic testing, the NIH GDS policy raised some questions. The relationship between a clinical laboratory and an individual receiving testing through their laboratory is different than that of a researcher and a research participant. Although researchers often have close contact with their participants and are required to obtain consent for all aspects of the study, including future research, most clinical laboratories have never interacted with their patients and often do not even have basic contact information. Without a previous relationship or even a method of contact, it is difficult for a clinical laboratory to recontact patients and obtain consent for data sharing efforts.

Following the release of the NIH GDS policy, ClinGen worked with the National Human Genome Research Institute (NHGRI) and ClinVar to consider the NIH GDS policy in the context of sharing interpreted variants from clinical genetic testing and to outline the data elements that might reasonably be included in a de-identified submission of variant-level data to ClinVar. The goal of this work is to encourage best practices for GDS of clinically obtained results while promoting the protection of the individuals tested.

## THE NIH GDS POLICY IN THE CONTEXT OF CLINICAL VARIANT-LEVEL DATA SHARING

De-identified variant-level information obtained by laboratories during the course of fee-for-service clinical testing may be submitted to ClinVar without the explicit consent of the patients in whom these variants were observed. Although not subject to the NIH GDS policy because submissions involve variant-level information and not large-scale human genomic data, the working group proposed that clinical submitters should still carefully consider the types of data that are appropriate to be shared in the absence of consent for data sharing and under what circumstances consent is recommended. The general framework presented here is intended for any submitter to inform discussion with institutional or country-specific approval bodies in regards to clinical de-identified variant-level data sharing with ClinVar and similar public repositories.

## INFORMATION NOT SUITABLE FOR SUBMISSION TO ClinVar

In general, certain information should never be submitted to ClinVar, including any of the 18 patient identifiers included in the Safe Harbor method of de-identification outlined in the Health Insurance Portability and Accountability Act (HIPAA) (patient name, date of birth, medical record number, etc.) (Standards for Privacy of Individually Identifiable Health Information 2002) or any other personal identifying information. Additionally, large genomic data sets (e.g., a VCF file from a whole-genome- or exome-sequencing study, all variants identified in a large gene panel, a cytogenomic microarray raw data file) should not be submitted to ClinVar. We recognize that there are studies in existence that do make this type of information publicly available at the participants’ request, such as the Personal Genome Project (Church 2005; Lunshof et al. 2008), but participants in these types of studies undergo a rigorous consent process to ensure that they understand the potential consequences of data sharing. For databases such as ClinVar that are unable to verify the level of consent any given individual has undergone, accepting such information would be inappropriate. ClinVar explicitly states in their submission guidance materials that they do not accept this type of information (https://www.ncbi.nlm.nih.gov/clinvar/docs/submit/).

## FRAMEWORK ON SHARING DE-IDENTIFIED VARIANT-LEVEL INFORMATION WITH PUBLICLY AVAILABLE DATABASES WHEN DIRECT PATIENT CONSENT WAS NOT SOUGHT

**Figure 1.**
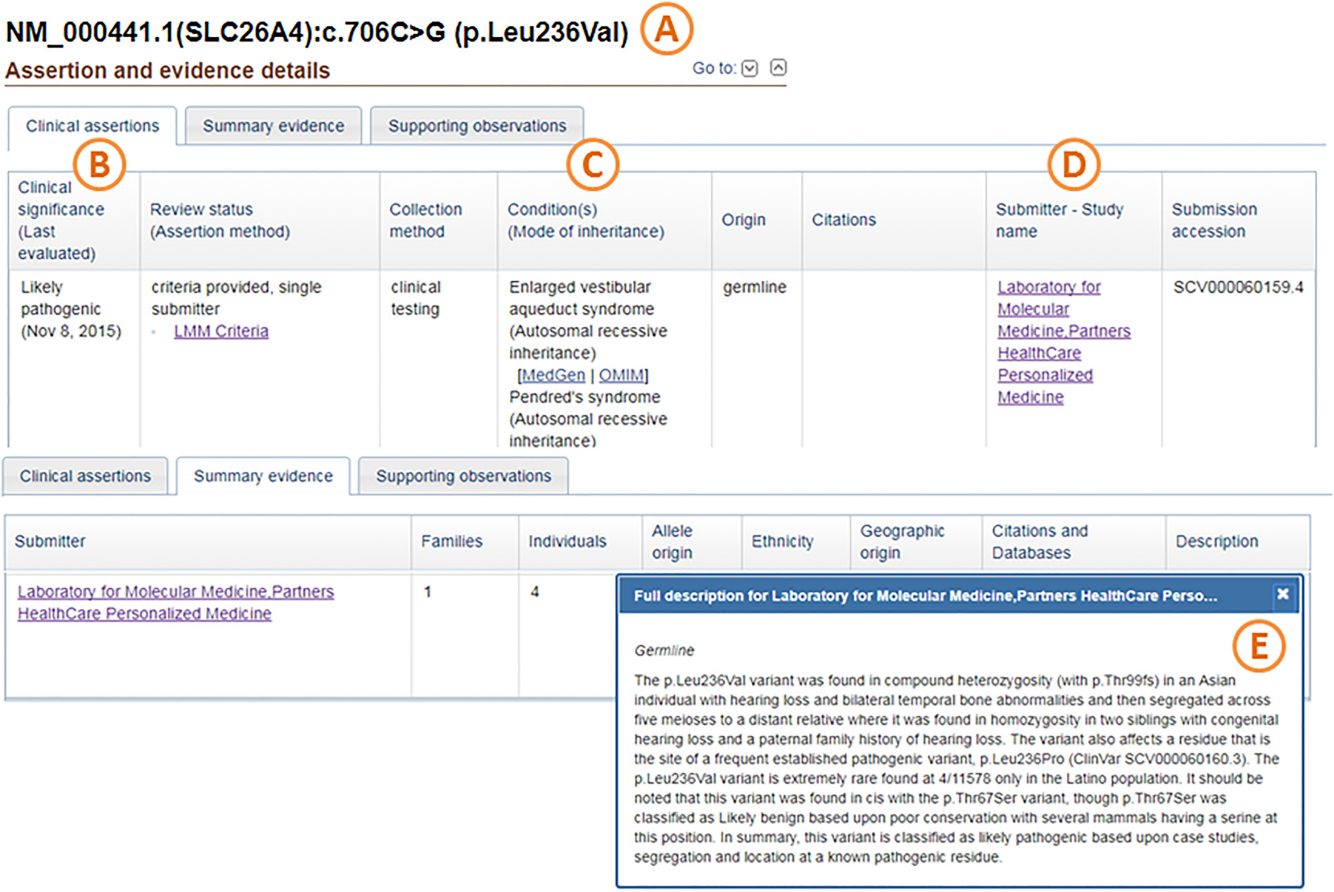
ClinVar display of data elements appropriate for public data sharing without explicit consent. (*A*) Standardized description of variant; (*B*) submitter's clinical significance assertion; (*C*) condition and inheritance pattern on which the submitter based the pathogenicity assertion; (*D*) name of submitter; (*E*) evidence on which the submitter based the clinical significance assertion. Selected views from ClinVar variation display for variation ID 43565 (https://www.ncbi.nlm.nih.gov/clinvar/variation/43565/ Accessed September 2017).

When direct patient consent for data sharing was not sought, which is often the case for genomic data generated through clinical testing, summary variant-level information may still be shared with ClinVar. Information submitted may include those elements listed in Table 1. Representation of these elements in ClinVar is displayed in Figure 1.

**Table 1.** Data elements appropriate for public data sharing without explicit consent

A. Valid description of variant (HGVS expression, genomic coordinates, and copy number, etc.)
B. Clinical significance assertion
C. Condition and inheritance pattern on which the assertion was based, if applicable
D. Name of clinical testing laboratory
E. Evidence on which the assertion was based including Published evidence A summary of one or more case observations Information on additional variants observed in individual patients as it relates to the assertion Experimental evidence

As clinical testing laboratories often receive samples for testing from around the world, and are not necessarily limited to only receiving samples from a local population, naming the submitting clinical laboratory is not believed to put individual privacy at substantial risk—one cannot assume with certainty the location of an individual based on the location of the laboratory performing their testing. Benefits of identifying the clinical testing laboratory include the ability of other professionals to contact the laboratory with questions about a variant interpretation, as well as the ability of the laboratory to participate in variant interpretation discrepancy resolution efforts with other submitting laboratories as a quality assurance activity (Garber et al. 2016; Harrison et al. 2017).

The description of the variant, the clinical significance assertion, and the condition on which the assertion was based are all properties of the variant relevant to the interpretation, not any individual patient. The assertion is usually associated with a condition and inheritance pattern, regardless of whether any individual patient has ever been diagnosed with that condition. For example, Variant X is pathogenic for Condition Y, inherited in an autosomal recessive manner. This does not imply that any of the individuals in whom this variant was observed are affected with Condition Y; information on the individual's other allele would be necessary to determine this, given the inheritance pattern of this condition. In the case of autosomal dominant conditions, the relationship between assertion condition and patient affected status may be more readily implied, although this does not necessarily reveal which features of a condition a given individual may have or may be at risk for developing.

The evidence on which the assertion is based can include, but is not limited to, a summary of available literature, summary of genetic, computational, and functional analyses published or performed internally, and/or a summary of the laboratory's case-level experience with the variant. A summary of case-level experience can include the number of individuals in whom the variant has been identified, as well as a summary and counts of the phenotypic and demographic features of those individuals. This information is typically a generalized description. In scenarios when a variant has been observed multiple times, a group description, in which no one individual can be discerned from the others in the group, can be used. For example: “This laboratory has observed this variant in five individuals: four males, one female, ranging in age from 18 to 62 yr. All cases had a clinical diagnosis of hypertrophic cardiomyopathy: Three had conduction system disease and two were reported to have onset in infancy.”

It should be noted that some variants (including, but not limited to, copy-number variants [CNVs]) may only have been observed in a single individual. This aspect alone does not preclude sharing of these unique variants and their associated phenotype(s) in databases such as ClinVar. Many clinically significant variants identified in clinical testing will only ever be observed once by a laboratory; choosing not to submit a given variant until a laboratory has accumulated additional cases may result in the variant never being shared (Rehm et al. 2015). These types of variants, the ones most often associated with disease pathogenicity, are some of the most valuable to share to improve interpretation; we argue that the potential harm from not sharing rare variants (potentially resulting in erroneous classifications from others) outweighs the risk of individual re-identification. However, one should be conservative with the amount of distinguishing information shared. We recommend that submitters consider using Human Phenotype Ontology (HPO) (Kohler et al. 2017) codes to describe phenotypes observed in tested individuals and to report only the phenotypic information necessary to provide other professionals a reasonable overview of the case. For example, in the case of an individual with a unique CNV and developmental delay and dysmorphic features, it may not be necessary to list each specific feature; listing only a few features relevant to the interpretation such as “hypertelorism” and “low-set ears” or the umbrella term “facial dysmorphism” may convey the minimum necessary information. Professionals interested in this case would be able to contact the submitting laboratory for more specific information if desired. Additionally, sensitive information such as HIV status, unique information such as an isolated ethnic group, or any information irrelevant to the interpretation of the case should be excluded from this type of sharing.

Furthermore, when making assertions on variants, it is often important to have access to information on additional variants observed in individual patients as it relates to the assertion of the variant being described. For example, a laboratory may want to note that the reason Variant A was considered benign for a dominant disorder is because the patient had a known pathogenic variant in *trans* in the same gene in which biallelic pathogenic variants are lethal. Other examples include reporting the variant in *trans* for a recessive condition or both components of an unbalanced translocation. The working group felt that it would be appropriate to share this information given its relevance to the interpretation of the original variant. In most instances, these data may only include disclosure of one additional variant; submitters should exercise caution when reporting numerous other variants observed in an individual, as each piece of additional information can increase the individual's vulnerability to re-identification.

## JUSTIFICATION FOR DATA ELEMENTS INCLUDED IN VARIANT-LEVEL SHARING

The variant-level data described here refers to aggregate information from individuals in whom the variant has been observed and may include a group description of these individuals, as described above. None of the 18 patient identifiers outlined in the HIPAA (Standards for Privacy of Individually Identifiable Health Information 2002) are included in the accepted data elements outlined in the policy.

Published cases of re-identification (Gymrek et al. 2013) of individuals with genetic information in publicly accessible databases and theoretical approaches to privacy breaches have been described (Benitez and Malin 2010; Atreya et al. 2013; Cai et al. 2015; Shringarpure and Bustamante 2015). However, these instances involved databases that included either full genomic data sets or sets of short tandem repeats on the Y chromosome, types of information that are not accepted in ClinVar. As such, there is little risk of re-identification through submission to ClinVar using such approaches.

To date, submissions from clinical testing laboratories represent the highest percentage (>80%) of variants in ClinVar. Because clinical testing laboratories offer testing for numerous conditions to clients all over the world, ClinVar includes information on a heterogeneous group of people, not limited to any one country, ethnic group, or disease population. This aspect of the data set would theoretically limit the effectiveness of attacks using identified resources for a specific population, such as voter registration records. Additionally, ClinVar does not accept full genomic data sets and does not link multiple variants from the same individual (except in cases where the interpretation of one variant affects another, as described above). Thus, the effectiveness of attacks based on the ratio of presence/absence of multiple alleles from an individual would be limited (Shringapure and Bustamante 2015).

Although the working group concluded that the risk of re-identification of an individual in ClinVar is low using current methodology, it is not impossible. One scenario in which re-identification may occur is when a patient can self-identify given the detailed data included in an entry. We believe this possibility poses little harm to the patient other than surprise if they were previously unaware of a laboratory's data sharing practices. For this reason, we encourage laboratories to make policies around data sharing with ClinVar clear. It should be noted that in some cases self-identification can be helpful. Anecdotally, we are aware of patients using ClinVar to discover information on other patients with variants they harbor, and knowing an entry represents their own data prevents time spent attempting to track down an erroneous second case. Similar to self-identification, the possibility of identification will be higher for a user with detailed knowledge of the particular case (such as a relative or a health-care provider). However, for the general population, we consider the sharing of de-identified variant-level information, as described here, to be of no more than minimal risk of identification to the tested participants.

## WHEN CONSENT MAY BE NEEDED: INDIVIDUAL-LEVEL DATA SHARING

As described above, a laboratory may submit a group description of the individuals in whom it has observed a variant without explicit consent. However, there are times when individually describing each member of that group would provide useful information. The ability to distinguish which features or confounding factors were present or absent in each individual in a group is helpful when trying to determine the effects of particular genetic variants. In this scenario, which we will refer to as “individual-level” data sharing, the individuals are distinguishable from one another, but no overtly identifiable information is being shared publicly. For example: “This laboratory has observed this variant in three individuals: one male, Hispanic, 18 yr old, with intellectual disability and autism; one female, Asian, 6 yr, with seizures and intellectual disability; and one male, African, 12 yr, with intellectual disability.” Increasing the distinguishing features of an individual theoretically makes them more vulnerable to potential re-identification efforts. Because of this potentially increased vulnerability, we posit that, when possible, the appropriate action would be to obtain informed consent before sharing this type of individual-level data with publicly available resources, such as ClinVar.

The working group recognizes the delicate balance between variant-level sharing of a variant only observed once by a laboratory (i.e., a private variant or “*n* of 1” case) and individual-level data sharing. As described in the previous section, we believe the potential harm of not sharing a rare variant observation outweighs the risk of individual re-identification and recommend sharing such cases with ClinVar as outlined, even in the absence of explicit consent. Sharing such information is still in line with the specifications of the Safe Harbor de-identification method and in accordance with the 2017 updates to the Common Rule, which reaffirmed the notion that data de-identified by this standard did not require consent for use (Federal Policy for the Protection of Human Subjects 2017). Although the individual-level information described above also conforms to these standards, we strongly promote, when possible, obtaining informed consent before delineating detailed individual-level data in public repositories.

To promote consent for individual-level data sharing and facilitate the adoption of this practice by clinical testing laboratories, ClinGen has developed a one-page consent form and supplemental online video for broad GDS, freely available at https://www.clinicalgenome.org/share (ER Riggs, DR Azzariti, A Niehaus, S Goehringer, EM Ramos, LL Rodriguez, BM Knoppers, HL Rehm, CL Martin, in prep.). The materials are consistent with the intent of the NIH GDS Policy and include all applicable elements discussed within NHGRI's Informed Consent Resource (https://www.genome.gov/27565449/the-informed-consent-resource/).

## COMPARISON TO OTHER CLINICAL DATA SHARING CONSENT POLICIES

Recently, another GDS consortium, the Matchmaker Exchange (MME), proposed a tiered consent policy for sharing data in the context of genomic matchmaking (Dyke et al. 2017). The goal of genomic matchmaking is to identify multiple individuals with a similar phenotype and the same candidate gene to build up evidence for gene–disease causality. In line with our framework on variant-level submission to ClinVar, MME has recommended that matchmaking with gene candidates can occur without explicit patient consent, concluding that this is consistent with one of the intended goals of clinical care, to provide a diagnosis.

## CONCLUSION

The question of when consent is needed for data sharing of clinical genomic information does not have a straightforward answer. No general policy currently exists that explicitly describes the threshold for requiring consent for sharing clinical genomic data for broad access. Ideally, all parties involved in the clinical genetic testing process would be informed of data sharing practices and written consent would be routinely obtained; however, clinical laboratories often do not have a relationship with their patients needed to obtain consent, and clinicians may not have the resources to facilitate consent. As such, we have defined a framework to support data sharing through ClinVar and similar open access databases, with minimal risk of re-identification, that does not rely on explicit individual consent in most cases. We recommend all laboratories clearly document their variant and individual-level data sharing practices and make this information readily available to patients and providers (e.g., on their website) to provide transparency and raise awareness (ACMG Board of Directors 2017).

Given the established partnership between ClinGen and ClinVar (https://www.clinicalgenome.org/about/about-the-clingen-and-clinvar-partnership/), this working group specifically focused on considerations for data sharing with ClinVar. However, the considerations outlined here may be applicable to other public repositories of genomic and phenotypic data around the world. We encourage databases supporting similar levels of variant- and individual-level data sharing to examine and adopt these principles as appropriate.

In summary, we have defined best practices for submitters by outlining elements of variant-level data that can be shared with ClinVar in the absence of explicit consent. For sharing more detailed individual-level information, obtaining explicit written consent is advised. One must balance the need to contribute to the growing genomics knowledge base, a critical step in improving the interpretation of genomic variants, with an individual's right to privacy and autonomy.

## ADDITIONAL INFORMATION

### Acknowledgments

We thank the many ClinVar submitters for sharing their data as well as Elyse Galloway and Kate Saylor, formerly of the NHGRI Division of Policy, Communications, and Education.

### Author Contributions

D.R.A, E.R.R., A.N., L.L.R., E.M.R., M.J.L., C.L.M., and H.L.R. drafted the framework for submitting de-identified variant-level information to ClinVar. B.K. is responsible for NCBI resources including ClinVar. All authors contributed to the manuscript.

### Funding

Research reported in this publication was supported by the National Human Genome Research Institute (NHGRI), in conjunction with additional funding from the Eunice Kennedy Shriver National Institute of Child Health and Human Development (NICHD) under award number U41HG006834. ClinVar is supported by the Intramural Research Program of the National Library of Medicine, National Institutes of Health.

### Competing Interest Statement

The authors have declared no competing interest.

## References

[AZZARITIMCS002345C1] ACMG Board of Directors. 2017 Laboratory and clinical genomic data sharing is crucial to improving genetic health care: a position statement of the American College of Medical Genetics and Genomics. Genet Med 19: 721–722. 28055021 10.1038/gim.2016.196

[AZZARITIMCS002345C2] American Medical Association. 2013 Genome Analysis and Variant Identification Policy D-460.971.

[AZZARITIMCS002345C3] Atreya RV, Smith JC, McCoy AB, Malin B, Miller RA. 2013 Reducing patient re-identification risk for laboratory results within research datasets. J Am Med Inform Assoc 20: 95–101. 22822040 10.1136/amiajnl-2012-001026 PMC3555327

[AZZARITIMCS002345C4] Benitez K, Malin B. 2010 Evaluating re-identification risks with respect to the HIPAA privacy rule. J Am Med Inform Assoc 17: 169–177. 20190059 10.1136/jamia.2009.000026 PMC3000773

[AZZARITIMCS002345C5] Cai R, Hao Z, Winslett M, Xiao X, Yang Y, Zhang Z, Zhou S. 2015 Deterministic identification of specific individuals from GWAS results. Bioinformatics 31: 1701–1707. 25630377 10.1093/bioinformatics/btv018 PMC4443672

[AZZARITIMCS002345C6] Church GM. 2005 The personal genome project. Mol Syst Biol 1: 2005.0030. 10.1038/msb4100040 PMC1681452 16729065

[AZZARITIMCS002345C7] Department of Health and Human Services. 2002 Standards for Privacy of Individually Identifiable Health Information 45 CFR Parts 160 and 164. 10.3109/15360288.2015.1037530 26095483

[AZZARITIMCS002345C8] Department of Health and Human Services. 2017 Federal Policy for the Protection of Human Subjects 45 CFR Part 46. 10.3109/15360288.2015.1037530 26095483

[AZZARITIMCS002345C9] Dyke SOM, Knoppers BM, Hamosh A, Firth HV, Hurles M, Brudno M, Boycott KM, Philippakis AA, Rehm HL. 2017 “Matching” consent to purpose: the example of the Matchmaker Exchange. Hum Mutat. doi: 10.1002/humu.23278. PMC5669800 28699299

[AZZARITIMCS002345C10] Garber KB, Vincent LM, Alexander JJ, Bean LJ, Bale S, Hegde M. 2016 Reassessment of genomic sequence variation to harmonize interpretation for personalized medicine. Am J Hum Genet 99: 1140–1149. 27843123 10.1016/j.ajhg.2016.09.015 PMC5097932

[AZZARITIMCS002345C11] Gymrek M, McGuire AL, Golan D, Halperin E, Erlich Y. 2013 Identifying personal genomes by surname inference. Science 339: 321–324. 23329047 10.1126/science.1229566

[AZZARITIMCS002345C12] Harrison SM, Dolinsky JS, Knight Johnson AE, Pesaran T, Azzariti DR, Bale S, Chao EC, Das S, Vincent L, Rehm HL. 2017 Clinical laboratories collaborate to resolve differences in variant interpretations submitted to ClinVar. Genet Med 19: 1096–1104. 28301460 10.1038/gim.2017.14 PMC5600649

[AZZARITIMCS002345C13] Kirkpatrick BE, Riggs ER, Azzariti DR, Miller VR, Ledbetter DH, Miller DT, Rehm H, Martin CL, Faucett WA, ClinGen Resource. 2015 GenomeConnect: matchmaking between patients, clinical laboratories, and researchers to improve genomic knowledge. Hum Mutat 36: 974–978. 26178529 10.1002/humu.22838 PMC4575269

[AZZARITIMCS002345C14] Kohler S, Vasilevsky NA, Engelstad M, Foster E, McMurry J, Ayme S, Baynam G, Bello SM, Boerkoel CF, Boycott KM, 2017 The Human Phenotype Ontology in 2017. Nucleic Acids Res 45: D865–D876. 27899602 10.1093/nar/gkw1039 PMC5210535

[AZZARITIMCS002345C15] Landrum MJ, Lee JM, Benson M, Brown G, Chao C, Chitipiralla S, Gu B, Hart J, Hoffman D, Hoover J, 2016 ClinVar: public archive of interpretations of clinically relevant variants. Nucleic Acids Res 44: D862–D868. 26582918 10.1093/nar/gkv1222 PMC4702865

[AZZARITIMCS002345C16] Landrum MJ, Lee JM, Riley GR, Jang W, Rubinstein WS, Church DM, Maglott DR. 2014 ClinVar: public archive of relationships among sequence variation and human phenotype. Nucleic Acids Res 42: D980–D985. 24234437 10.1093/nar/gkt1113 PMC3965032

[AZZARITIMCS002345C17] Lunshof JE, Chadwick R, Vorhaus DB, Church GM. 2008 From genetic privacy to open consent. Nat Rev Genet 9: 406–411. 18379574 10.1038/nrg2360

[AZZARITIMCS002345C18] National Institutes of Health. 2014 Genomic Data Sharing 79 FR 51345.

[AZZARITIMCS002345C19] National Society of Genetic Counselors. 2015 Clinical Data Sharing.

[AZZARITIMCS002345C20] Rehm HL, Berg JS, Brooks LD, Bustamante CD, Evans JP, Landrum MJ, Ledbetter DH, Maglott DR, Martin CL, Nussbaum RL, 2015 ClinGen—the Clinical Genome Resource. N Engl J Med 372: 2235–2242. 26014595 10.1056/NEJMsr1406261 PMC4474187

[AZZARITIMCS002345C21] Shringarpure SS, Bustamante CD. 2015 Privacy risks from genomic data-sharing beacons. Am J Hum Genet 97: 631–646. 26522470 10.1016/j.ajhg.2015.09.010 PMC4667107

